# Supplementary material for: Pharmacologic inhibition of protein phosphatase-2A achieves durable immune-mediated antitumor activity when combined with PD-1 blockade
Source: Nat Commun. 2018 May 29;9:2126. doi: 10.1038/s41467-018-04425-z (PMC5974350; doi:10.1038/s41467-018-04425-z)
Supplement: Supplementary file 1 — Supplementary Information [file 41467_2018_4425_MOESM1_ESM.pdf]

Pharmacologic inhibition of protein phosphatase-2A achieves durable immune-mediated antitumor activity when combined with PD-1 blockade

Ho et al.

Supplementary Information

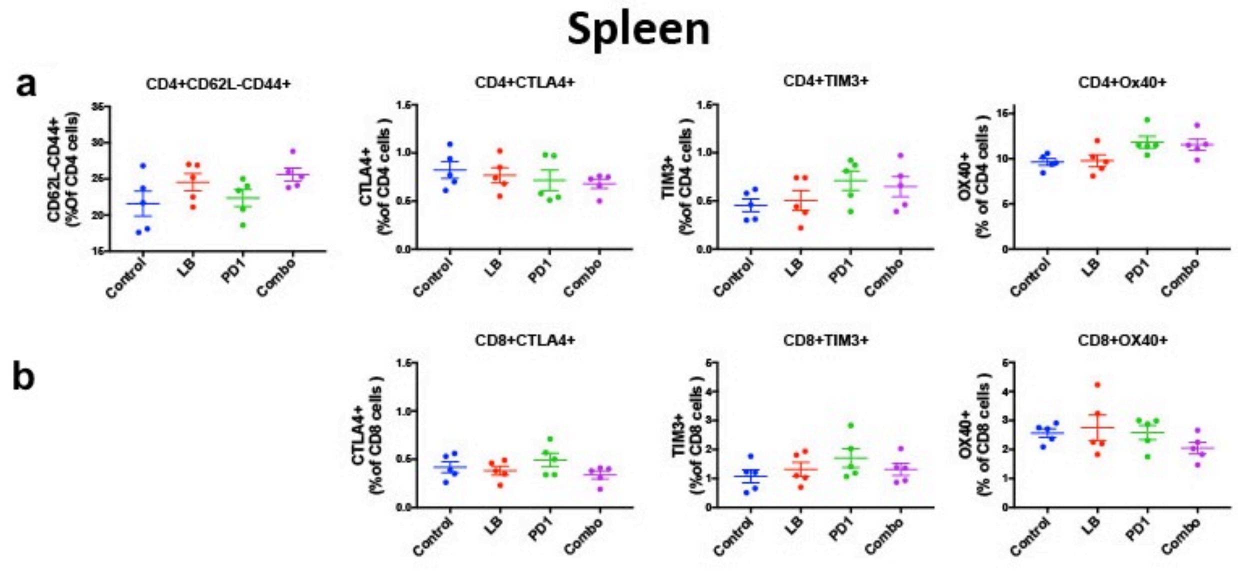

Supplementary Figure. 1. Flow cytometric analysis of activation and immune checkpoint markers of (a) CD4+ and (b) CD8+ lymphocytes in the spleen of mice receiving LB-100 and/or aPD-1 treatment.

(a) In CD4+ T cells, unlike CD8+ T cells, there was no change in expression of CD62L-CD44+ expression. There was also no change in expression of immune check point markers: CTLA4, TIM3 and Ox40.

(b) In CD8+ T cells, there was no change in expression of immune check point markers: CTLA4, TIM3 and Ox40.

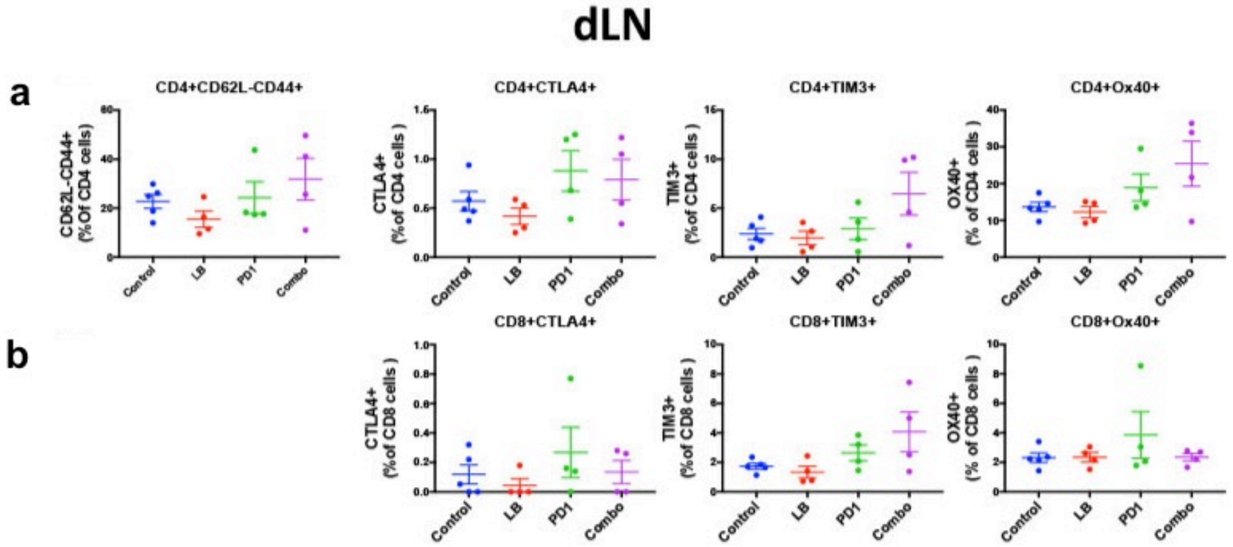

Supplementary Figure 2. Flow cytometric analysis of activation and immune checkpoint markers of (a) CD4+ and (b) CD8+ lymphocytes in the draining lymph node (dLN) of mice receiving LB-100 and/or aPD-1 treatment.

(a) In CD4+ T cells, unlike CD8+ T cells, there was no change in expression of CD62L-CD44+ expression. There was no change in expression of immune check point markers: CTLA4, TIM3 and Ox40.

(b) In CD8+ T cells, there was no change in expression of immune check point markers: CTLA4, TIM3 and Ox40.

*$P < 0.05$, **<$P<0.01$ (one way ANOVA with Tukey's multiple comparison test). Error bars depict SEM.

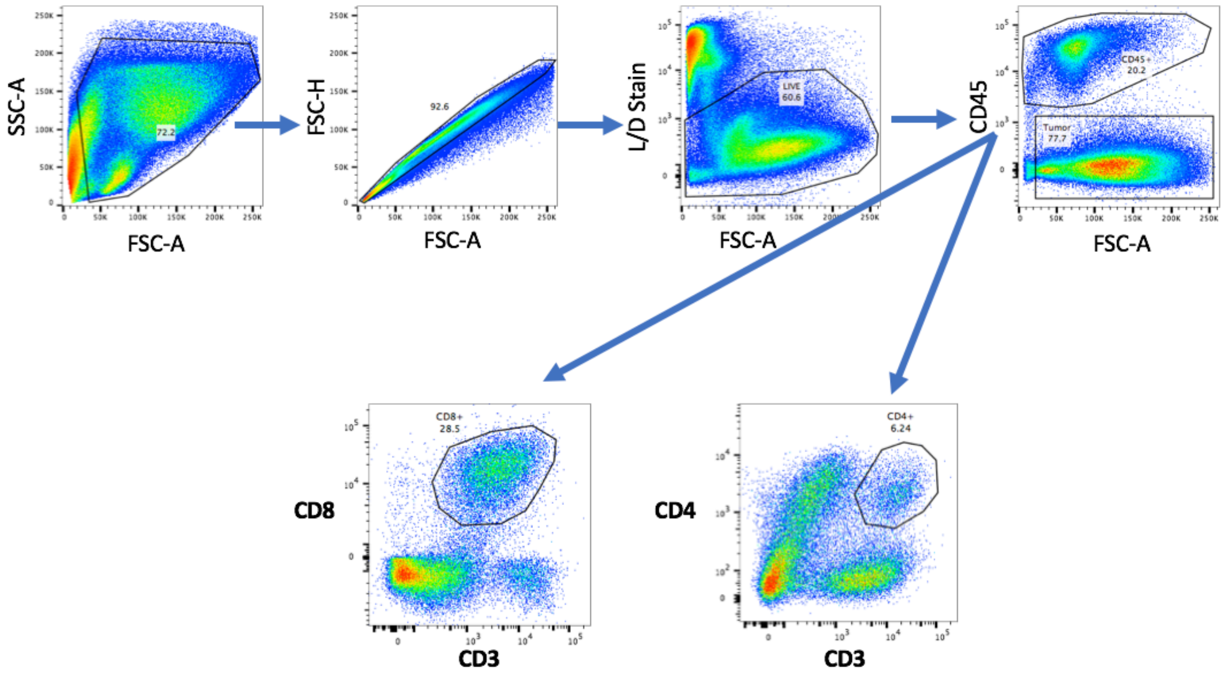

Supplementary Figure 3. Gating strategy for flow cytometric analysis of tumor infiltrating lymphocytes. We first used SSC-FSC gate to exclude non-cellular debris, followed by exclusion of duplets by FSC-H-FSA-A gate. Fixable live-dead (L/D) stain was used to exclude dead cells. Live cells were then gated based on expression of CD45+ pan leukocyte marker. CD45- cells were considered as tumor cells. CD45+ cells were then phenotyped further based on CD3, CD8, CD4 expression. CD45+CD3+CD8+ cells were gated as CD8+ lymphocytes, while CD45+CD3+CD4+ cells were gated as CD4+ lymphocytes. Further, staining of the CD4+ and CD8+ subsets were then performed as indicated in the text.

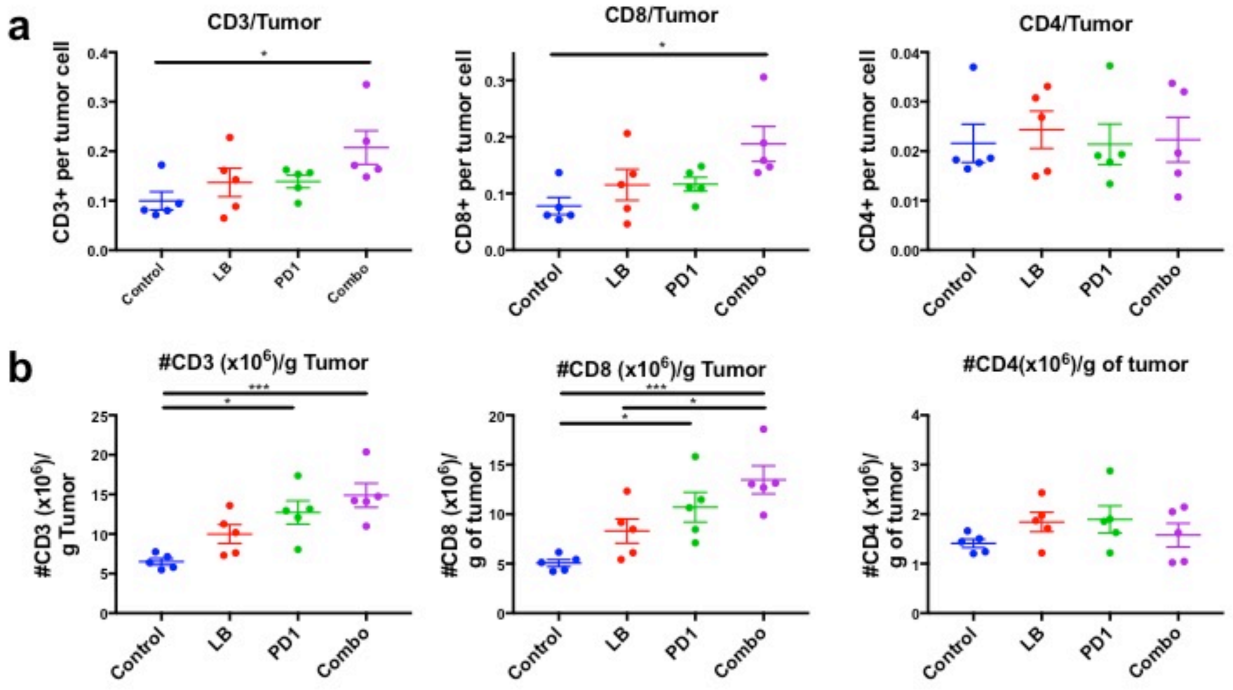

Supplementary Figure 4. aPD-1 and LB-100 combination increased infiltration of CD8+ lymphocytes in tumors. BALB/c mice were inoculated with CT26 tumor cells (0.5×10^6). When tumor volume reached 50-100 mm^3 (day 0), they were randomized into four treatment groups as described in the main text: PBS control (blue), LB-100 (red), aPD-1 (green), combination (purple). After two treatments, on day 3, tumors were harvested and analyzed by flow cytometry. (a) The ratios of CD3+, CD8+, and CD4+ cells to CD45- tumor-resident cells were shown for each treatment group. There was an increase in CD3/tumor and CD8/tumor ratios in the combination group compared to control, while there was no change in CD4/tumor ratio.
(b) The number of CD3+, CD8+ and CD4+ cell per gram of tumor weight were shown for each treatment group. A similar trend was seen as in (a), but there were significant differences in CD3+ and CD8+ per gram tumor in aPD-1 treated group alone compared to control. There was a trend of further increase in CD3+ and CD8+/tumor for combination treatment, but there was no statistical significance. *$P < 0.05$, ***<P<0.001 (one way ANOVA with Tukey's multiple comparison test). Error bars depict SEM.

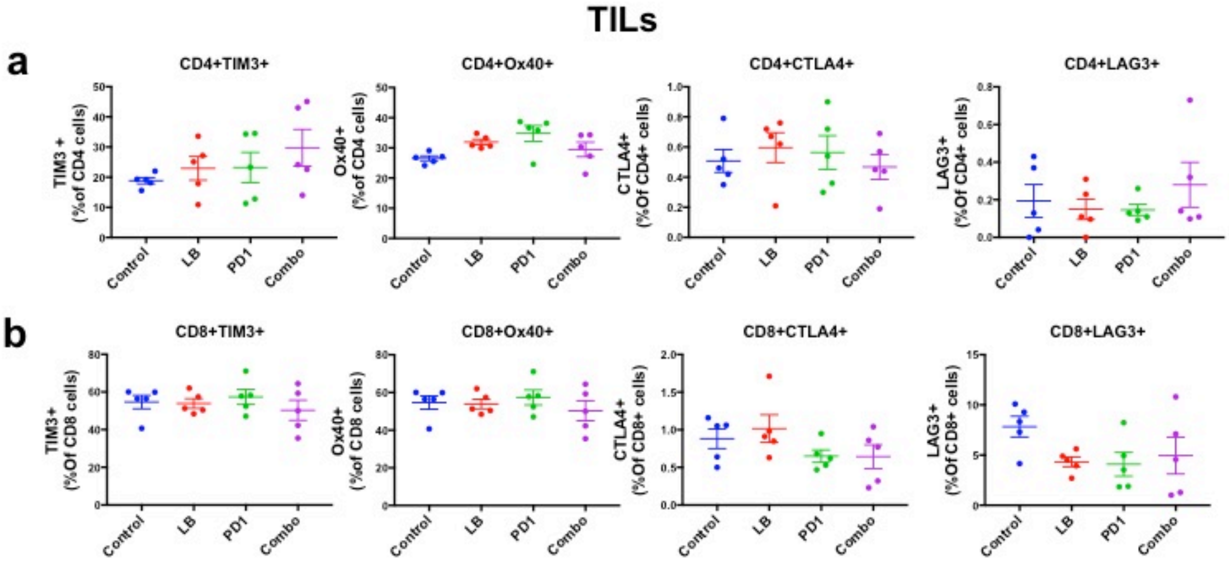

Supplementary Figure 5. Flow cytometric analysis of and immune checkpoint markers of (a) CD4+ and (b) CD8+ lymphocytes in tumors of mice receiving LB-100 and/or aPD-1 treatment.

(a) In CD4+ T cells, there was no change in expression of immune checkpoint markers: TIM3, Ox40, CTLA4 and LAG3.

(b) In CD8+ T cells, there was no change in expression of immune checkpoint markers: TIM3, Ox40, CTLA4 and LAG3.

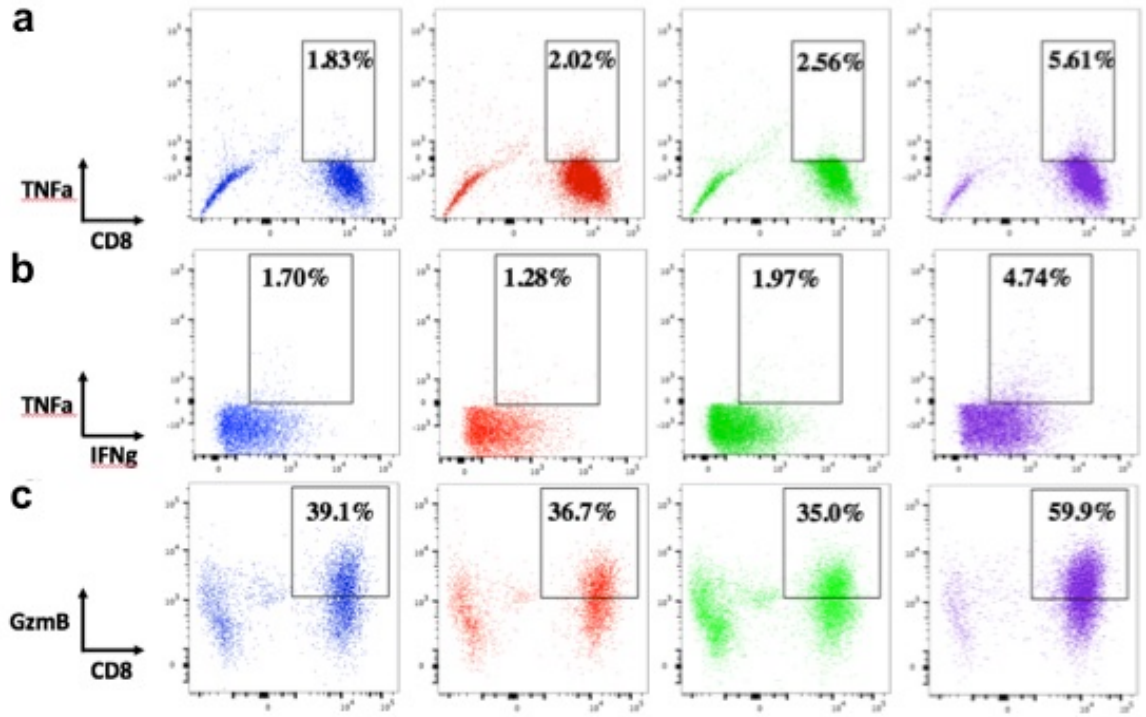

Supplementary Figure 6. aPD-1 and LB-100 combination increased effector function of tumor infiltrating CD8+ lymphocytes. Tumor cell suspensions were similarly harvested as above. Cells were then stimulated ex-vivo with PMA/Ionomycin and protein transport inhibitor for 5 hours before stained and fixed for flow cytometry analysis. Representative flow cytometry plot showing increased TNF-α+ (a), TNF-α+ IFN-γ+ double positive (b) and GranzymeB+ (c) CD8 tumor infiltrating T-cells. Percentage displayed are of total (a) CD3+, (b) CD8+ and (b) CD3+ cells.

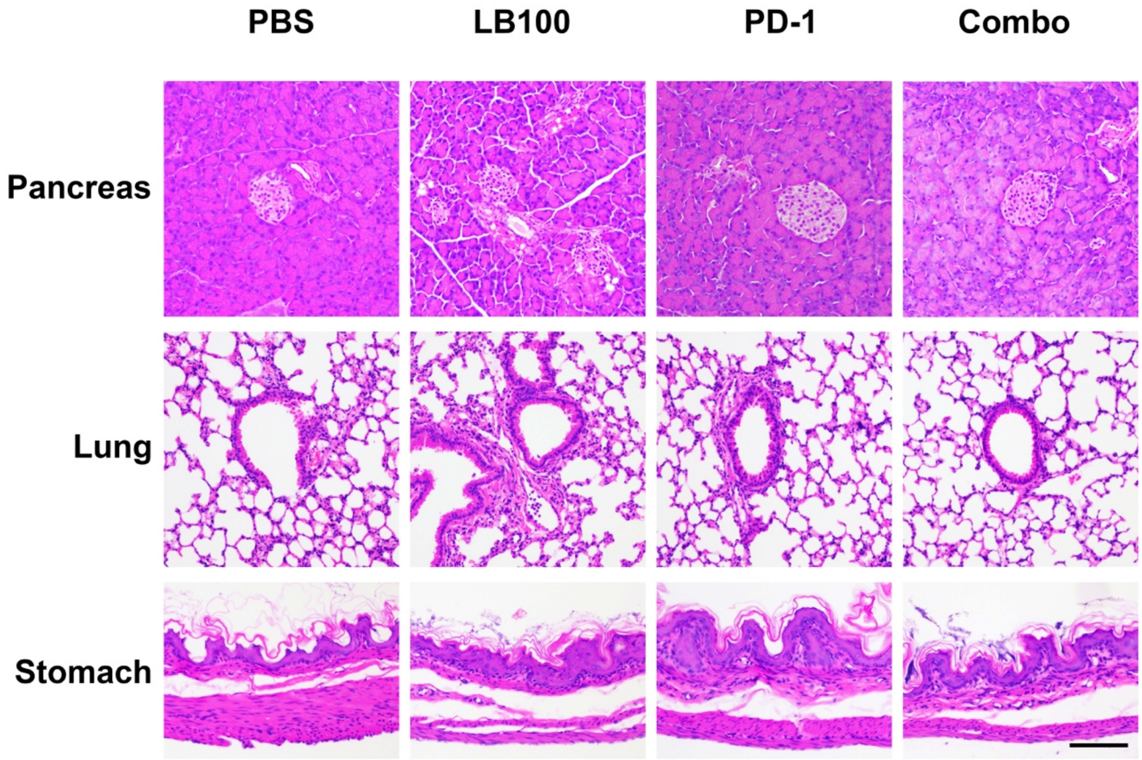

Supplementary Figure 7. aPD-1 and LB-100 combination treatment did not develop signs of autoimmunity in multiple organs. C57BL/6 mice were randomized into four treatment groups. $2.5x10^5$ B16F10 cells were inoculated 2 days after initiation of treatment subcutaneously in the right thoracic flank. Mice were treated every two days until survival endpoint. At the time of sacrifice, organs were harvested for fixation and staining. Representative images of hematoxylin-and-eosin staining of the pancreas, lung and stomach of each treatment group (n = 2 per group). Scale bars, 100 µm.

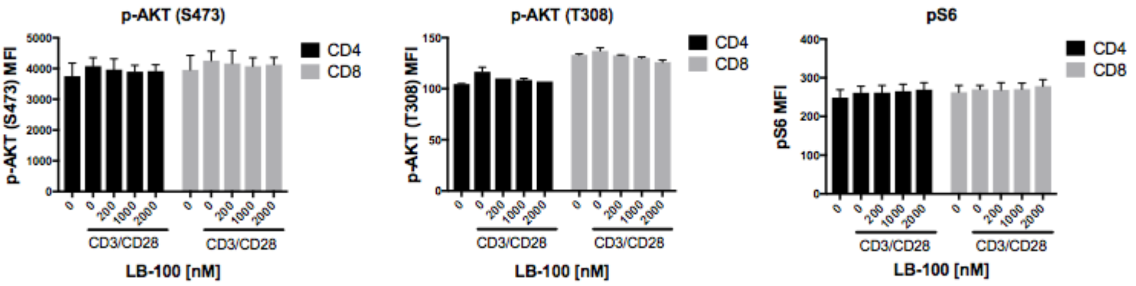

Supplementary Figure 8. AKT and mTORC signaling after 30 minutes of stimulation. CD3 T cells were stimulated using immobilized anti-CD3 (10 µg ml^{-1}) and soluble anti-CD28 (2 µg ml^{-1}). Flow cytometry analyzing AKT phosphorylated at Thr308 (p-AKT(T308)), Ser473 (p-AKT(S473)) or phosphorylated S6 (p-S6) after 30 minutes of stimulation in presence of LB-100 dose titration. (one way ANOVA with Tukey's multiple comparison test). Data are from one experiment representative of two independent experiments with similar results. Error bars depict SEM.

Main Pharmacokinetic Parameters of LB-100 in SD Rats following IV Administration

Analyte	Dose of LB-100 mg kg^{-1}	Tissue	$T_{1/2}$ (h)	T_{max} (h)	C_{max} (ng ml^{-1} or ng g^{-1})	AUC_{last} (ng h ml^{-1} or ng h g^{-1})	AUC_{INF} (ng h ml^{-1} or ng h g^{-1})	MRT (h)
LB-100	0.5	Brain	/	0.25	17.4	10.8	/	/
		Liver	0.78	0.25	586	758.6	902.2	1.17
		Plasma	0.31	0.25	1110	695.8	706.0	0.45
	1.0	Brain	1.67	0.25	33.9	35.3	72.5	2.68
		Liver	0.79	0.25	1371	3526.5	3537.7	1.51
		Plasma	0.99	0.25	2383	1923.5	2830.2	1.57
	1.5	Brain	1.93	0.25	43.5	125.5	140.8	2.57
		Liver	2.01	1	2548	9081.0	10449.1	2.90
		Plasma	2.20	0.25	3664	7399.6	8641.4	2.82

Supplementary Table 1. Pharmacokinetic parameters of LB-100 in rats. Following single IV administration of LB-100, tissue concentration of LB-100 in plasma, liver and brain tissue concentrations were determined by liquid chromatography-mass spectrometry. LB-100 could pass to a limited extent through blood-brain barrier (BBB) following iv administration at 0.5, 1.0 and 1.5 mg kg^{-1}. The mean Cmax in plasma was 1110~3664 ng ml^{-1}. The mean Cmax in liver and brain were 586~2548 ng kg^{-1} and 17.4~43.5 ng kg^{-1}, respectively. AUClast in plasma was 695.8~7399.6 ng h ml^{-1}, with 758.6~9081.0 ng h g^{-1} in liver and 10.8~125.5 ng h g^{-1} in brain, respectively. T1/2 in plasma, liver and brain were 0.31~2.20 h, 0.78~2.01h and 1.67~1.93 h, respectively.

$T_{1/2}$ – Half-life
T_{max} – Time to maximum concentration
C_{max} – Maxiumum concentration
AUC_{last} - Area under the time-concentration curve (0 to the last measurable concentration)
AUC_{last} - Area under the time-concentration curve (0 to the infinity)
MRT - Mean residence time

Main Pharmacokinetic Parameters of Endothall in SD Rats following IV Administration

Analyte	Dose of LB-100 mg kg^{-1}	Tissue	$T_{1/2}$ (h)	T_{max} (h)	C_{max} (ng ml^{-1} or ng g^{-1})	AUC_{last} (ng h ml^{-1} or ng h g^{-1})	AUC_{INF} (ng h ml^{-1} or ng h g^{-1})	MRT (h)
Endothall	0.5	Brain	/	/	/	/	/	/
		Liver	10.1	0.25	349	2598	3095	7.90
		Plasma	6.65	0.25	577	564	828	2.96
	1.0	Brain	/	/	/	/	/	/
		Liver	6.10	0.25	1425	6673	7370	6.14
		Plasma	7.06	0.25	1230	2487	2750	4.38
	1.5	Brain	/	/	/	/	/	/
		Liver	4.57	0.25	2964	18434	18850	4.54
		Plasma	6.25	0.25	1178	4476	4730	4.57

Supplementary Table 2. Pharmacokinetic parameters of endothall in rats. Following single IV administration of LB-100, tissue concentration of LB-100 metabolite, endothall, in plasma, liver and brain were determined by liquid chromatography-mass spectrometry. Endothall was detectable in plasma and liver samples following single iv administration of LB-100 at 0.5, 1.0 and 1.5 mg kg^{-1}, and the concentrations in plasma and liver increased with dose level of LB-100, whereas endothall was not detectable in brain samples. The mean Cmax in plasma and liver were 577-1230 ng ml^{-1} and 349-2964 ng ml^{-1}, respectively. AUClast in plasma and liver were 546-4476 ng h ml^{-1} and 2598-18434 ng h g^{-1}, respectively. T1/2 in plasma and liver were 6.25-7.06 h and 4.57-10.1h, respectively.

$T_{1/2}$ – Half-life
T_{max} – Time to maximum concentration
C_{max} – Maxiumum concentration
AUC_{last} - Area under the time-concentration curve (0 to the last measurable concentration)
AUC_{last} - Area under the time-concentration curve (0 to the infinity)
MRT - Mean residence time
